# Interpretable deep learning for personalized energy expenditure prediction using ECG and acceleration signals in incremental exercise

**DOI:** 10.1038/s41598-025-20195-3

**Published:** 2025-10-16

**Authors:** Yingzhe Song, Zhen Wang, Hongxing Wang, Gang Sun, Bingnan Gong, Fangfang Zhang

**Affiliations:** 1 Institute of Artificial Intelligence in Sports, Capital University of Physical Education and Sports, Beijing, 100191 China; 2 Department of Rehabilitation, School of Medicine, Chongqing University Three Gorges Hospital, Chongqing University, Chongqing, 404100 China; 3 Xinjiang Key Laboratory of Neurological Disorder Research, The Second Affiliated Hospital of Xinjiang Medical University, Urumqi, 830000 Xinjiang China; 4 Institute of Physical Education and Training, Capital University of Physical Education and Sports, Beijing, 100191 China; 5 Department of Anesthesiology, Xiangya Hospital, Central South University, National Clinical Research Center for Geriatric Disorders, Xiangya Hospital, Central South University, Changsha, 410000 China

**Keywords:** Computer science, Scientific data, Biomedical engineering

## Abstract

Energy expenditure (EE) assessment is crucial in both sports science and health management. However, current EE prediction models often overlook individual differences and lack dynamic correlation analysis between multi-modal data and EE. Building upon previous research, this study proposes a personalized dynamic-static feature fusion framework, which integrates two types of information to improve energy expenditure (EE) prediction during incremental load exercise: dynamic signals (physiological signals recorded continuously during exercise, such as tri-axial acceleration and electrocardiography [ECG]) and static physiological metrics (stable individual traits measured at rest, such as BMI, body-fat percentage, resting heart rate, and resting oxygen uptake [VO_2_]). These two feature sets were combined through a hybrid convolutional neural network (CNN) and long short-term memory (LSTM) neural network architecture. CNN layers extract local temporal patterns from dynamic signals, and LSTM layers model temporal dependencies over longer intervals. The model prediction performance was evaluated using root mean square error (RMSE), coefficient of determination (R²), mean absolute error (MAE) and Bland-Altman plots, and the results show that the CNN + LSTM model significantly outperforms both the traditional autoregressive (AR) linear model and the LSTM model that uses only a single modality (acceleration or ECG). Analysis of feature values and SHAP values revealed that accelerometer features played a dominant role in EE prediction during moderate-to-high intensity exercise. As exercise intensity increased, the contribution of ECG features gradually increased, with ECG features dominating during high-intensity exercise, demonstrating the complementary effect and dual contribution of these two types of features in EE prediction at different exercise intensities. This study demonstrates that personalized dynamic-static feature fusion can effectively predict EE during incremental exercise tests and analyzes the dynamic changes in the contribution of different features across different intensity ranges, providing a theoretical basis and methodological reference for related research.

## Introduction

Energy expenditure (EE) measurement and estimation are important in sports science and health care. Athletes’ physical load during training or competition can be better understood by tracking EE, which helps with injury prevention, athletic performance evaluation, and training program optimisation. Real-time EE monitoring makes it easier for people in healthy or sub-healthy states to implement efficient dietary and exercise plans, reducing the health risks related to energy intake and expenditure imbalances. Accurate EE measurement greatly improves the specificity and effectiveness of intervention strategies in populations with metabolic syndrome, overweight, or obesity^1^. During physical activity or at rest can be measured using direct or indirect calorimetry. Since its introduction in 1955, the doubly labelled water (DLW) method has been considered the gold standard for assessing EE under free-living conditions. However, its high cost and inability to provide real-time monitoring limit its practical application^2^. Metabolic gas analysers, regarded as one of the gold standards, estimate EE by measuring oxygen consumption and carbon dioxide production. Although they enable real-time monitoring during exercise, their application is limited due to the need for heavy face masks and restricted usage scenarios.

Wearable sensors are now a popular method for measuring EE during daily activities or exercise in order to overcome these limitations. Accelerometers, for instance, are used to detect changes in body acceleration and to create estimation models based on the dose-response relationship between acceleration and EE^3^. Heart rate (HR) sensors measure HR using photoplethysmography (PPG) or electrocardiography (ECG) and construct estimation models based on either linear or nonlinear relationships between EE and HR. Accelerometers based on micro-electromechanical system (MEMS) sensors detect body movements along three orthogonal axes the vertical, coronal, and sagittal axes and convert these changes into digital signals^4^. The accuracy of accelerometer signals in estimating EE has been confirmed by numerous studies^5^. Accelerometer signals, however, lose their usefulness for precise EE estimation as exercise intensity and movement complexity rise^6^. During submaximal exercise, HR, which measures the intensity of the internal load, has a linear relationship with metabolic rate and oxygen consumption^7^. Numerous factors, such as fitness level, age, height, weight, body composition, and baseline status, influence the slope of the relationship between HR and EE^8–10^. During moderate-to-high-intensity activities, HR increases linearly with oxygen consumption^11^. Some studies have created an S-shaped curve that depicts the relationship between EE and HR by using logistic models to account for individual variability^12^. However, research investigating and validating the relationship between EE and HR during maximal intensity exercise is still lacking.

With the continuous development of integrated sensors, wearable devices have enabled the synchronous acquisition of multisource heterogeneous data, combining physiological signals with acceleration signals. Meanwhile, the widespread application of machine learning algorithms and neural network models has provided new approaches for EE prediction. To tackle EE classification and regression tasks, for example, support vector machines, decision trees, random forests, and artificial neural networks have been used^13,14^.

Nevertheless, traditional regression algorithms usually depend on manually created features that are taken from predetermined time frames, failing to account for the ongoing changes in metabolic demands that occur during physical activity. In order to overcome this restriction, some research has suggested processing time-series acceleration signals using long short-term memory (LSTM) networks, which allows for activity classification and EE estimation while taking into consideration the temporal dependencies of EE^15^.

In order to create EE prediction models under slow gait conditions, other research has combined data from inertial sensors, surface electromyography (sEMG), and HR with LSTM and convolutional neural networks (CNNs), providing more dependable and ergonomic solutions^16^. Additionally, model fitting and predictive performance have been further enhanced by combining time-series networks with deep learning techniques like CNNs. By combining physiological and acceleration signals with environmental factors like food intake and ambient temperature, some studies have built customised EE prediction models based on CNN - LSTM frameworks, and they have shown exceptional predictive accuracy^17^.

Although time-series neural network models have achieved notable success in predicting EE, several limitations remain. First, most current studies rely solely on HR and its derived indicators for physiological feature analysis, with limited exploration of raw electrocardiogram (ECG) signals. Some studies have demonstrated that simple time-domain and frequency-domain features extracted from R interval data are highly correlated with exercise intensity, yet few have applied ECG signals directly for EE prediction^18,19^. Second, individual differences are frequently disregarded. EE during exercise is strongly influenced by important physiological factors like body mass index (BMI) and resting metabolic rate, but these individual differences have not been adequately taken into account by current models^20^. Additionally, the majority of current research concentrates on EE prediction during fixed-pattern or low-to-moderate-intensity activities, with little research done on complex and high-intensity movements. Previous studies have confirmed that accelerometer signals and HR exhibit distinct response characteristics at different exercise intensities. Although neural networks integrate features from multiple sensor signals during model construction, they seldom conduct a quantitative analysis of feature contributions across varying intensity levels, leading to a lack of interpretability in EE predictions.

In summary, this study proposes a personalised dynamic-static feature fusion framework based on prior research, constructing an EE prediction model using individual-specific differences and acceleration and ECG signals collected during incremental load exercises. The predictive performance of various models under different feature combinations is evaluated. Furthermore, Shapley Additive Explanations (SHAP) values are employed to analyse both global and local feature importance, and the changes in feature contributions across different exercise intensity levels are investigated, providing theoretical insights for future research on energy expenditure prediction.

## Methods

### Participants

For this study, 24 college students between the ages of 18 and 25 were chosen at random. To guarantee their capacity to safely finish the experimental protocol, each participant gave written informed consent prior to participation and underwent a pre-exercise health screening. The Capital University of Physical Education and Sports Ethics Committee gave its approval to all procedures, which were carried out in compliance with the Declaration of Helsinki (Approval No. 2024A098). Five participants’ data were insufficient because of equipment failures during the experiment, and as a result, they were not included in the final analysis.

### Experimental design

Each participant completed two experimental sessions: a resting test and an exercise test. The resting test was conducted in the early morning after an overnight fast, with participants lying in a supine position. Following the resting measurement, body weight and body fat percentage (BF%) were assessed, and body mass index (BMI) was calculated based on height and weight. The exercise test was performed on the same day after completing the resting measurements.

During the exercise test, participants performed an incremental treadmill protocol based on the RAMP protocol described in the ACSM’s Guidelines for Exercise Testing and Prescription. The test began at a speed of 3 km/h, with the running speed increasing by 0.5 km/h every 30 s. The test was terminated when participants met any of the following criteria: HR reaching 90% of the estimated maximal HR (calculated as 220 minus age), respiratory exchange ratio (RER) greater than 1.15, the rating of perceived exertion (RPE) went above 17, or a plateau in oxygen uptake was observed.

Prior to the exercise test, all participants performed a 5-minute dynamic stretching and jogging warm-up. To minimise the influence of environmental and physiological factors on EE, both resting and exercise tests were conducted in the same laboratory under controlled conditions: ambient temperature was maintained between 25 and 27 °C, and humidity was kept at approximately 50%. Participants were instructed to refrain from food intake within 12 h before the resting test and to avoid vigorous exercise, alcohol, or caffeine consumption within 24 h prior to the exercise test.

### Data collection

Body weight and body fat percentage (BF%) were measured using the INBODY-270 body composition analyzer. Resting oxygen consumption (RVO_2_) was recorded with the Schiller gas metabolism analyzer, and heart rate (HR) was measured using a Polar H10 heart rate monitor. During the exercise test, the Schiller gas metabolism analyzer was also used to continuously monitor oxygen uptake (VO_2_) and carbon dioxide production (VCO_2_) throughout the entire exercise protocol. Participants wore a face mask connected to the gas analyzer during testing. The gas analyzer was calibrated before each test session to ensure accurate oxygen and carbon dioxide measurements. EE was calculated using the validated Weir Eq. (2)^21^.

Acceleration and ECG signals were continuously recorded during the exercise test. Acceleration data were collected using a digital posture sensor from Shenzhen WitMotion Technology Co., Ltd. The sensor had a sampling range of 0.1 to 200 Hz, with a fixed sampling rate of 10 Hz for this study. It was worn on the non-dominant wrist, about 2–3 cm away from the wrist joint. ECG signals were recorded using the Polar H10 heart rate monitor, with a sampling frequency of 1 Hz and an accuracy error of less than 1 beat per minute (bpm). The device was positioned on the left chest, near the right side of the heart. The baseline physiological parameters recorded during the resting test are listed in Table 1.

**Table 1:** Baseline physiological parameters collected during the resting test.

Static features	Mean ± SD
Height(cm)	169.89 ± 8.42
Weight(kg)	63.41 ± 11.74
BMI(kg/m²)	21.71 ± 2.65
Bodyfat(%)	16.89 ± 6.68
Rest Heart(bpm)	59.74 ± 7.09
Rest VO_2_(ml/kg/min)	3.69 ± 0.76

### Model construction

The LSTM network, a type of RNN designed for dynamic time series, solves the vanishing gradient problem that occurs in traditional RNN training. By using memory cells and gating mechanisms, LSTM can selectively remember or forget past information, allowing it to capture or predict the current state based on changes in features from previous time steps. This enhances the network’s ability to model the complex dependencies within dynamic feature sequences. In this study, time series-based algorithms and networks were used to build models that reflect the dynamic variations in sequential data collected during exercise.

#### Data preprocessing

During the exercise tests, acceleration signals and ECG signals were collected, including acceleration data along the X, Y, and Z axes, and the time intervals between successive R waves (RR intervals). These time-series data exhibited noise and multiscale dynamic variations. To address missing values, the Local Outlier Factor (LOF) method was employed. LOF is a density-based outlier detection algorithm that identifies local anomalies by comparing the density of a point with that of its neighboring points^22^. Given that exercise intensity increased every 30 s according to the test protocol, the neighborhood size was set to 300 for processing acceleration signals and 30 for processing ECG signals. Outliers within the signals were filtered using a Kalman Filter, a recursive algorithm commonly used to estimate the state of dynamic systems in noisy environments^23^.

#### Feature extraction

Tri-axial acceleration signals and ECG signals respectively reflect external mechanical load and internal physiological responses. Recurrent Neural Networks (RNNs), such as LSTM, are capable of capturing sequential dependencies through weight sharing across time steps. However, the periodic characteristics of running-related signals are more distinctly represented in the frequency domain. Therefore, in this study, a 10-second time window was used. Frequency-domain features of the tri-axial acceleration signals were obtained using the Fast Fourier Transform (FFT), while ECG spectral features were derived through Power Spectral Density (PSD) analysis. The extracted features were designed to provide a comprehensive representation of both the periodicity of movement patterns and the regulation of physiological load. The dynamic time-series features and frequency-domain features are summarized in Table 2. A total of 22 dynamic features were extracted. For Acc signals, features such as signal energy, vector magnitude, and amplitude were calculated to characterize physical movement states. ECG features were derived based on heart rate variability (HRV) analysis to assess autonomic nervous system activity and physiological load. Accurate EE prediction relies on capturing the full spectrum of signals generated during exercise. Given that acceleration signals and ECG signals reflect external and internal loads, respectively, three combinations of input features were designed for model construction:

1. Acceleration signal features only;
2. ECG signal features only;
3. A combined input of both Acceleration and ECG features.

**Table 2:** Extracted dynamic time-series and frequency-domain features from acceleration and ECG signals.

Feature type	Features	Consept description
Acc	AccX, AccY, AccZ	Three-axis acceleration signals, which describe motion states in different directions
	VM (Vector Magnitude)	Scalar magnitude of tri-axial acceleration, which quantifies the overall intensity of movement, VM =
	AccX/Y/Z_TotalEnergy	Total energy along the X, Y, and Z axes and vector magnitude
	VM_TotalEnergy	
	AccX/Y/Z_MeanAmplitude	Mean amplitude of acceleration on the X, Y, and Z axes and vetor magnitude
	VM_MeanAmplitude	
ECG	R_Interval_Sec	Time between heartbeats; reflects HRV
	R_Interval_TotalPower	Overall HRV intensity
	ULFPower	Reflects chronic physiological regulation in the ultra-low-frequency range
	VLFPower	Reflects sympathetic nervous system activity in the very-low-frequency range
	LFPower	Represents combined sympathetic and parasympathetic modulation
	HFPower	Indicates parasympathetic activity; reflects respiratory sinus arrhythmia
	R_Interval_PeakFrequency	Frequency at which R-R interval spectral power reaches its maximum
	R_Interval_MeanPower	Mean spectral power of R-R intervals over a specified frequency range
	LF_HFRatio	Assesses the balance between sympathetic and parasympathetic activity
	HeartRate	Direct indicator of cardiac workload and physiological demand

#### Personalized dynamic-static feature fusion framework

In this study, a personalised dynamic-static feature fusion framework is proposed. The core idea is to separate and process the static features reflecting individual physiological characteristics from the dynamic time-series signals during the movement process, and then fuse the features after extracting them through different sub-networks in order to enhance the personalised recognition capability of energy consumption prediction. Dynamic and static features are input into the sub-networks separately, dynamic features are modelled using a time-series network to capture the short-term and long-term dependencies of the signals, and static features are fused with the modelled time-series features using a fully-connected layer with linear transformed mapping and finally input into a fully-connected regression layer. Based on the above model architecture, three different networks are selected for comparison:

1. Autoregressive linear model (AR): A baseline model using only dynamic features for EE prediction.
2. LSTM: Implements the personalized dynamic-static feature fusion framework, processing dynamic and static features through separate subnetworks and fusing them for final EE estimation.
3. CNN + LSTM: Also adopts the fusion framework, combining convolutional layers for local feature extraction with LSTM layers to model temporal dependencies.

AR linear models use only dynamic inputs as benchmarks. In the personalised feature fusion framework, dynamic signals are processed by either an LSTM encoder or a hybrid CNN + LSTM encoder. The LSTM-based variant captures temporal dependencies through sequential modelling, whereas the CNN + LSTM architecture first uses a convolutional layer to extract local temporal patterns and then captures long-term dependencies through the LSTM layer. Static physiological features (e.g., body composition and resting state measurements) are processed through separate fully connected sub-networks. The outputs of the dynamic and static branches are then connected and the EE is estimated through a final regression layer.

**Fig. 1:**
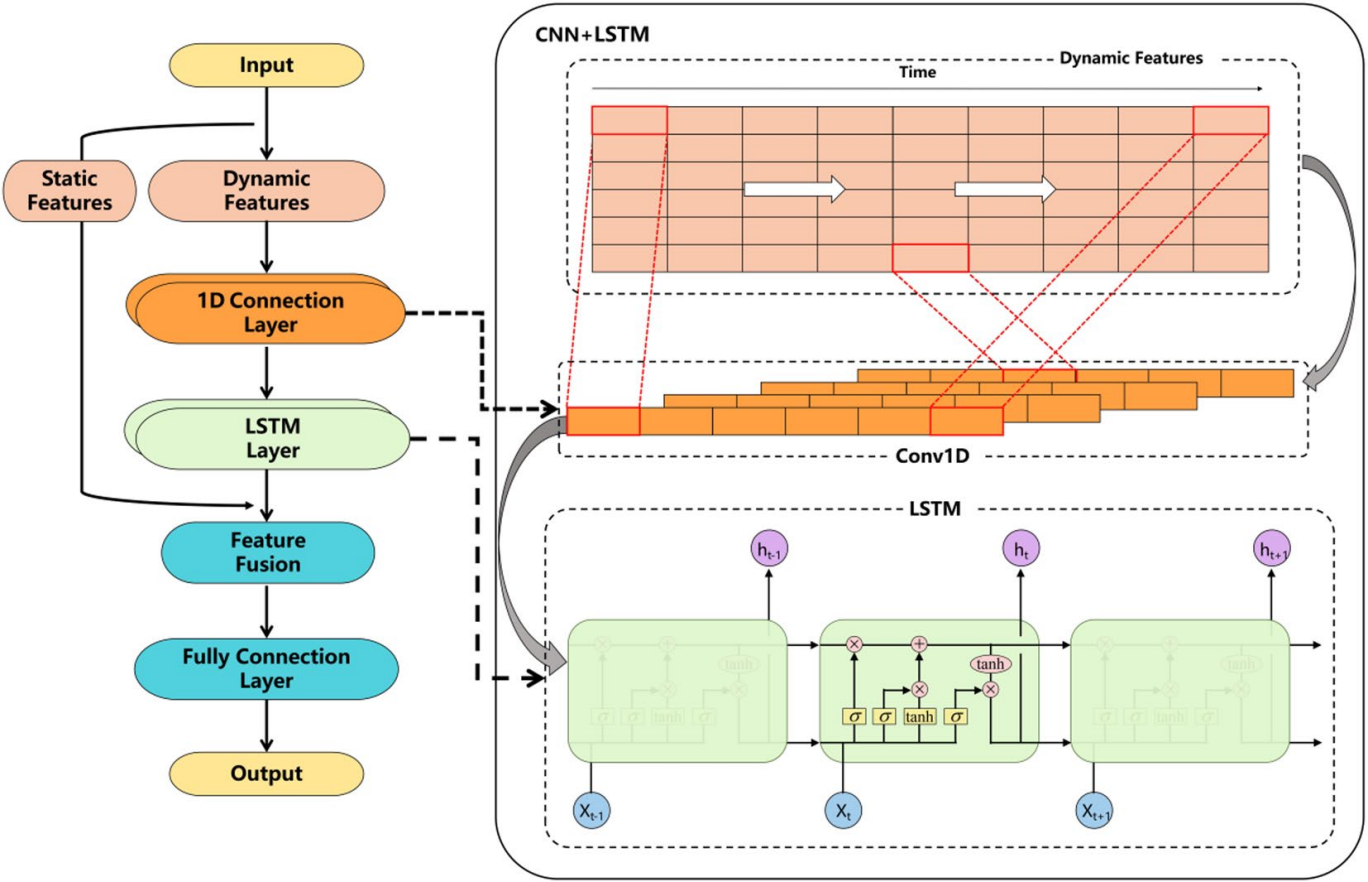
Personalised dynamic-static feature fusion framework based on CNN + LSTM.

As shown in Fig. 1, the CNN + LSTM model incorporates the structure of a convolutional neural network (CNN) and a long short-term memory network (LSTM), where the convolutional layer is used to identify local temporal trend changes in the input sequence, such as periodic movement fluctuations or transient changes in the signal. These locally encoded features are then passed to the LSTM layer, which uses its memory mechanism to maintain continuous modelling of the temporal context.CNN enhances the robustness of the model to local perturbations, whereas LSTM retains long term information about the historical state of the sequence, which is particularly crucial for tasks that rely on temporal changes such as energy consumption prediction. The structure has been widely used in human activity recognition, ECG signal analysis, and wearable device monitoring, demonstrating its effectiveness in modelling complex physiological processes.

#### Dataset partitioning and model evaluation approach

The extracted dynamic features were normalised before proceeding to model training. The metrics collected from the participants’ resting tests were used as static features. The time series model requires the data to be in the format of time step, number of samples and number of features. Considering the sampling frequency of EE is 0.1 Hz, the time step is set to 10. The number of dynamic features is 22, the number of static features is 6, and the number of samples is 1,323. Randomly splitting the dataset into training, validation and test sets may result in samples from the same individual appearing in both training and testing phases, potentially leading to data leakage. To minimize this risk, this study adopted a Leave-One-Subject-Out Cross-Validation (LOSO-CV) strategy. In each fold, data from one subject were used as the validation set, while data from the remaining subjects were used for training. Additionally, data from four participants were reserved as an independent test set and completely excluded from both training and cross-validation procedures to assess the model’s generalization ability. Since LOSO-CV produces averaged performance metrics across validation folds, the final model was retrained using the complete dataset from the 15 non-test subjects. This final model was then evaluated on the independent test set to determine its predictive performance on previously unseen individuals.

#### Implementation details and parameters

The main parameter for the construction of the AR benchmark model is the lag order p. In this study, the lag order is set to 10 considering that the time window of the dynamic time series is set to 10. In this study, the model hyperparameters are combined with preliminary small-scale pre-experiments and manual grid search to iteratively test the candidate hyperparameter combinations and gradually approach the optimal interval. The range of hyperparameters examined includes: the number of LSTM hidden cells (32, 64, 128, 256), Dropout ratio (0.1, 0.2, 0.3), and learning rate (0.1, 0.01, 0.001). For the CNN + LSTM model, the pairwise combinations of convolutional kernel size (3, 5, 7) and the number of convolutional channels (16, 32, 64) were also evaluated. The CNN pooling kernel size was fixed to 2, the step size was 1, the activation function was ReLU, and the number of neurons in the fully connected layer for final feature fusion was set to 32. The optimal configurations are: Hidden Unit 64, Dropout 0.1, Batch Size 32, Learning Rate 0.001, Convolutional Kernel Size 3, and Convolutional Channels 64/128. The model is trained using the Adam optimiser with a loss function of the Mean Squared Error (MSE), and 500 epochs per fold are trained in LOSO-CV. The model is trained using the Adam optimiser, with a loss function of Mean Square Error (MSE); 500 epochs per fold are trained in LOSO-CV, and 1,500 epochs of data from 15 subjects are finally integrated to evaluate the generalisation performance on an independent test set.

### Statistical analysis

The preprocessing of the data and the evaluation of the training of the models for this study were implemented in Excel 2013 and PyTorch 2.2.1. The performance of the model was evaluated using the Root Mean Square Error (RMSE), Mean Absolute Error (MAE) and Coefficient of Determination (R^2^) of the test set.

1 $$\:\text{RMSE=}\sqrt{\left\{\frac{\text{1}}{\text{n}}\sum\:_{\text{i}\text{=1}}^{\text{n}}\text{(}{\text{y}}_{\text{i}}\text{-}{\widehat{\text{y}}}_{\text{i}}{\text{)}}^{\text{2}}\right\}}$$

Where n is the total number of samples, $\:{\text{y}}_{\text{i}}$ and $\:{\widehat{\text{y}}}_{\text{i}}$ are the true value and predicted value of the i-th sample, respectively.

2 $${\text{MAE}} = \frac{1}{n}\mathop \sum \limits_{{i = 1}}^{n} \left| {y_{i} - \hat{y}_{i} } \right|$$

Calculate the absolute average value of the prediction error. Other symbols have the same meanings as in the RMSE Eq. 

3 $$R^{2} = 1 - \frac{{\mathop \sum \nolimits_{{i = 1}}^{n} (y_{i} - \hat{y}_{i} )^{2} }}{{\mathop \sum \nolimits_{{i = 1}}^{n} (y_{i} - \bar{y})^{2} }}$$

Where $\bar{y}$ is the mean of the true values, and the meanings of the other symbols are the same as in the RMSE equation.

The consistency of the model predictions was also assessed using Bland-Altman plots to calculate the systematic bias (Bias) and the limit of agreement (LoA). The significance of features was further interpreted globally and locally using the SHAP (Shapley Additive Explanations) library in Python.

## Results

### Model performance

We followed the personalized dynamic-static feature fusion framework and tested EE prediction performance with three models (AR, LSTM, CNN + LSTM) and three feature sets (Acc, ECG, Acc + ECG). The LOSO-CV results are presented in Table 3, and the test set results are shown in Table 4. The results show that using feature fusion and more complex models clearly improves accuracy. The 15-fold average performance metrics are 0.886 for R^2^, 0.080 for RMSE, and 0.062 for MAE, which are significant advantages over AR and LSTM models that use only unimodal input features. The prediction results on the independent test set further validate the generalisation performance of the model. The CNN + LSTM fusion model achieves an R^2^ of 0.906, an RMSE of 0.080, and an MAE of 0.049 on the independent test set, which indicates that the model has stable prediction results in the population of unseen subjects, and its generalisation ability is better than that of the AR and LSTM models.

**Fig. 2:**
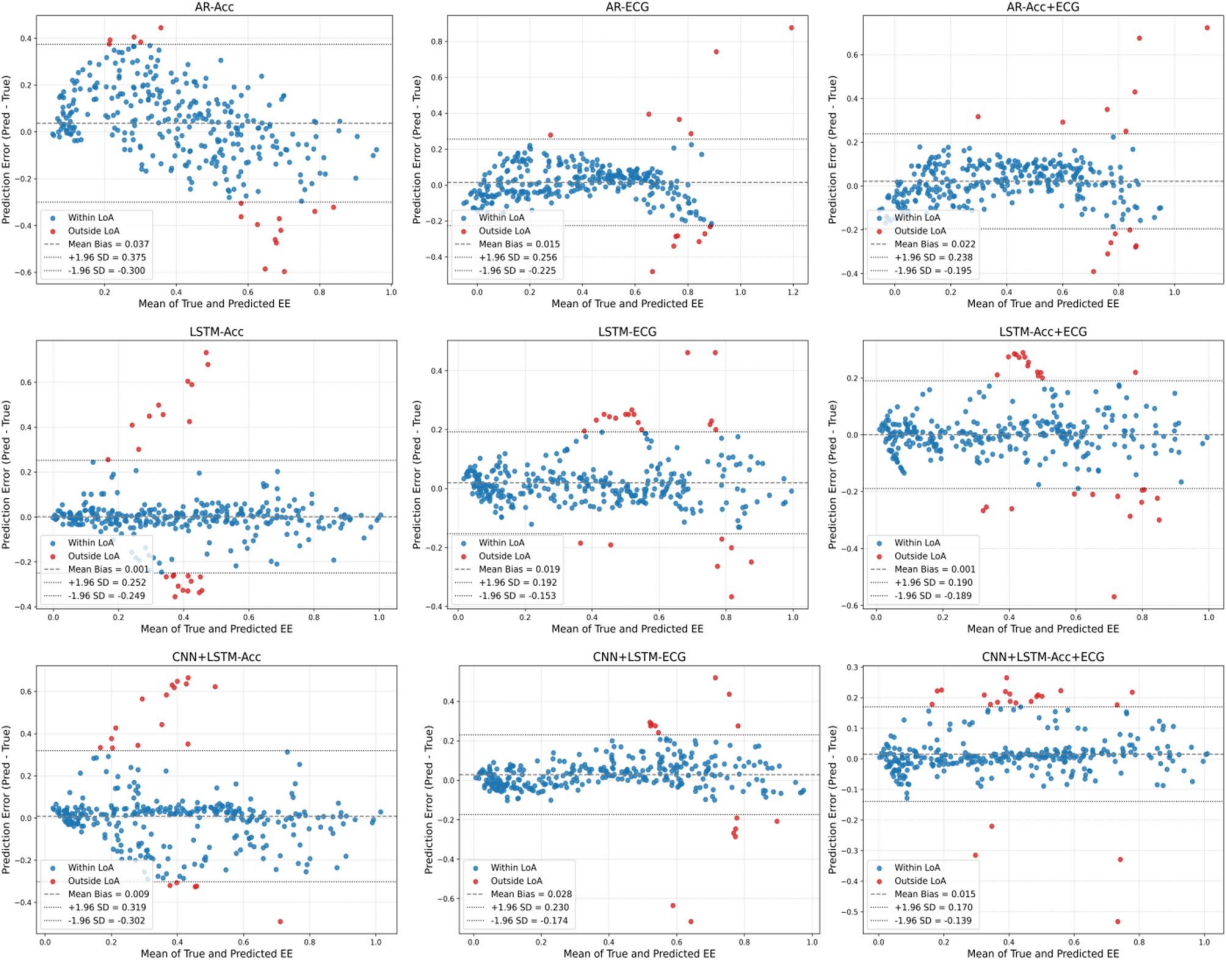
Analysis of Bland-Altman plots for different models.

The prediction error distribution of the AR model using only acceleration features is more discrete, with an average deviation of 0.037 and a 95% consistency interval of -0.300 to 0.375; the error is slightly reduced when ECG features alone are used, with a deviation of 0.015 and a consistency interval of -0.225 to 0.256; and the distribution of the error is further concentrated after fusion of the two types of features (Acc + ECG), with a deviation of 0.022 and a consistency interval of -0.195 to 0.238, indicating that feature fusion improves consistency performance. of 0.022 and a consistency interval of -0.195 to 0.238, indicating that feature fusion improves the consistency performance. In contrast, the prediction performance of the LSTM model is improved after the introduction of personalised static features, in which the prediction error of the acceleration feature only is significantly lower than that of the AR model; the error deviation of the ECG feature is 0.019, with a consistency interval of -0.153 to 0.192, which is further improved; and the error deviation of the fused feature is even smaller, only 0.001, with a consistency interval of -0.189 to 0.190, which shows that feature fusion and static feature incorporation significantly improve the consistency and stability of model prediction. The fusion of features and the inclusion of static features significantly improve the consistency and stability of model prediction. The CNN + LSTM model integrates the local feature extraction capability of the CNN layer and the LSTM layer’s advantage of long-term dependence on signal features, and the prediction error is further reduced. When only acceleration features are used, the error deviation is 0.009, with a consistency interval of -0.302 to 0.319; when only ECG features are used, the error deviation is 0.028, with a consistency interval of -0.174 to 0.230; when the two features are fused together, the error distribution is even closer, with an average deviation of 0.015, with a consistency interval of -0.139 to 0.170, and the overall error is closer to zero. The overall error is closer to zero. Fusing static features greatly improves the model’s ability to capture physiological differences between individuals and reduces individual prediction errors. As shown in Fig. 2.

**Table 3:** Average prediction performance of LOSO-CV.

Model	Feature	*R*^2^ (Mean ± SD)	RMSE (Mean ± SD)	MAE (Mean ± SD)
AR	ACC	0.567 ± 0.202	0.246 ± 0.080	0.192 ± 0.059
	ECG	0.791 ± 0.135	0.121 ± 0.04	0.10 ± 0.040
	ECG + ACC	0.747 ± 0.168	0.132 ± 0.048	0.105 ± 0.043
LSTM	ACC	0.530 ± 0.337	0.170 ± 0.086	0.130 ± 0.065
	ECG	0.867 ± 0.170	0.088 ± 0.058	0.071 ± 0.047
	ECG + ACC	0.855 ± 0.130	0.097 ± 0.050	0.076 ± 0.042
CNN + LSTM	ACC	0.617 ± 0.381	0.139 ± 0.096	0.105 ± 0.072
	ECG	0.870 ± 0.141	0.090 ± 0.053	0.072 ± 0.044
	ECG + ACC	0.886 ± 0.136	0.080 ± 0.056	0.062 ± 0.046

**Table 4:** EE model test set prediction performance.

Model	Feature	*R* ^2^	RMSE	MAE
AR	ACC	0.552	0.176	0.137
	ECG	0.779	0.124	0.084
	ECG + ACC	0.817	0.113	0.079
LSTM	ACC	0.523	0.182	0.123
	ECG	0.882	0.090	0.059
	ECG + ACC	0.865	0.0.97	0.063
CNN + LSTM	ACC	0.636	0.159	0.098
	ECG	0.835	0.107	0.068
	ECG + ACC	0.906	0.080	0.048

### Global feature importance analysis

**Fig. 3:**
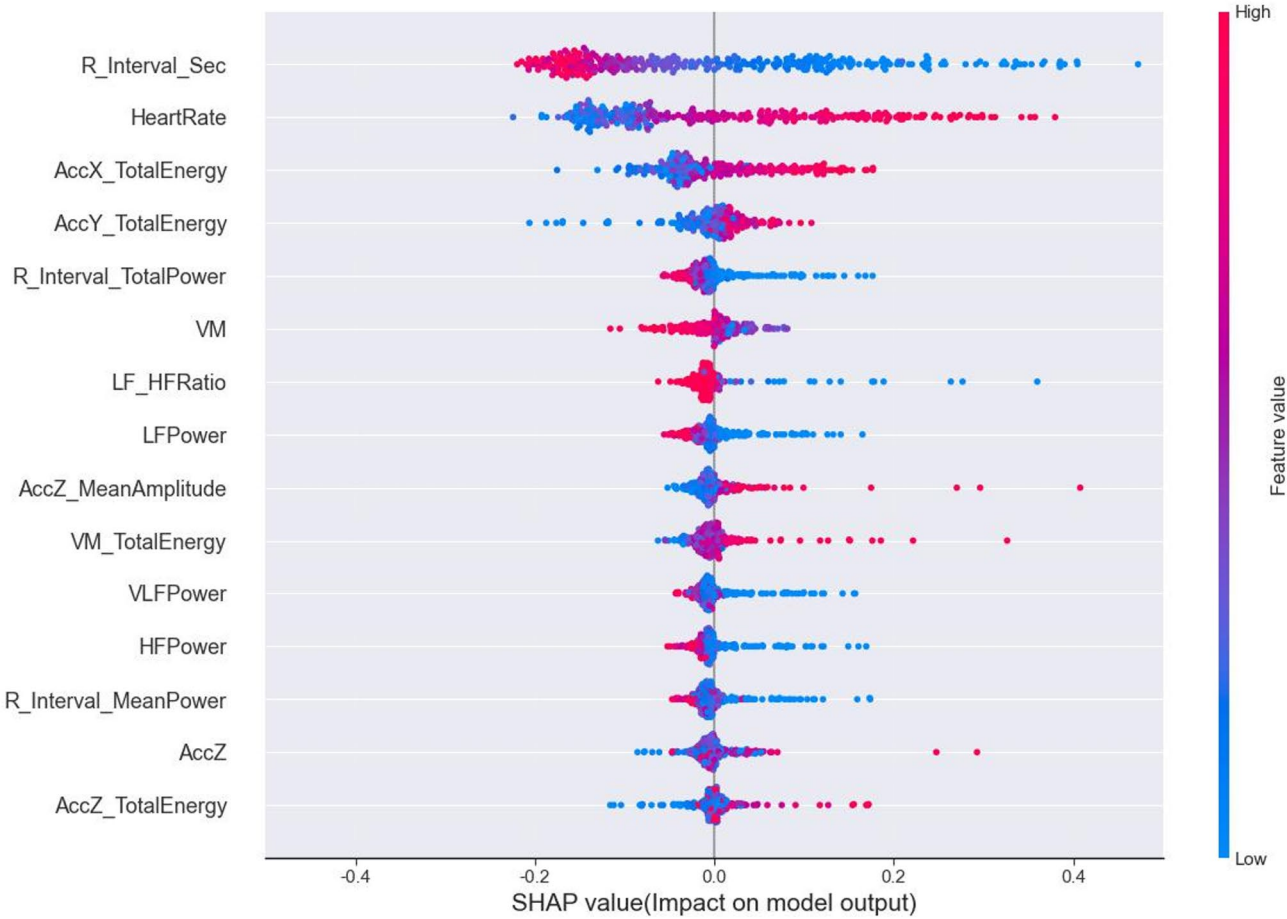
Summary of SHAP values for CNN + LSTM model.

The combination of dynamic features includes ECG and acceleration frequency domain features extracted using power spectral density and Fourier transform. The personalised dynamic-static feature fusion framework is able to accurately predict EE values, but the prediction model lacks the explanation of the internal mechanisms and the relationship between features. To further explore the feature contribution and importance of different feature combinations in personalised EE prediction, the personalised CNN + LSTM model fusing two types of dynamic features is selected for global and local SHAP value analysis in this study. Figure 3 demonstrates the magnitude and direction of the influence of the features with the top 15 ranked SHAP values on the EE output results, where the red colour represents higher feature values, the blue colour represents lower feature values, and the horizontal axis indicates the degree of influence on the EE prediction result.

SHAP analysis revealed that the ECG feature R_Interval_Sec had the most significant contribution to model prediction. R_Interval_Sec represents the duration between two consecutive R peaks in the ECG signal, representing one complete cardiac cycle. According to the SHAP results, an increase in R_Interval_Sec generally led to a decrease in predicted EE (indicated by negative SHAP values), while shorter R intervals (represented by blue points) were linked to higher predicted EE. This aligns with the physiological understanding that EE is positively correlated with HR. Other frequency-domain ECG features-such as R_Interval_TotalPower, LFPower, HFPower, and LF_HFRatio-also significantly influenced the model’s predictions, reflecting their role in indicating autonomic nervous system activity and internal physiological load.

Among the acceleration features, the frequency-domain features of triaxial acceleration (AccX_TotalEnergy, AccY_TotalEnergy, AccY_TotalEnergy, VM_TotalEnergy and AccZ_MeanAmpiltude), extracted using FFT, contributed notably to the model’s output. These features primarily captured trends in external loading during incremental exercise. Additionally, AccZ and VM further enhanced model performance. Indicating that it effectively captures dynamic changes across different directions and magnitudes of motion when estimating EE.

### Local feature importance analysis

**Fig. 4:**
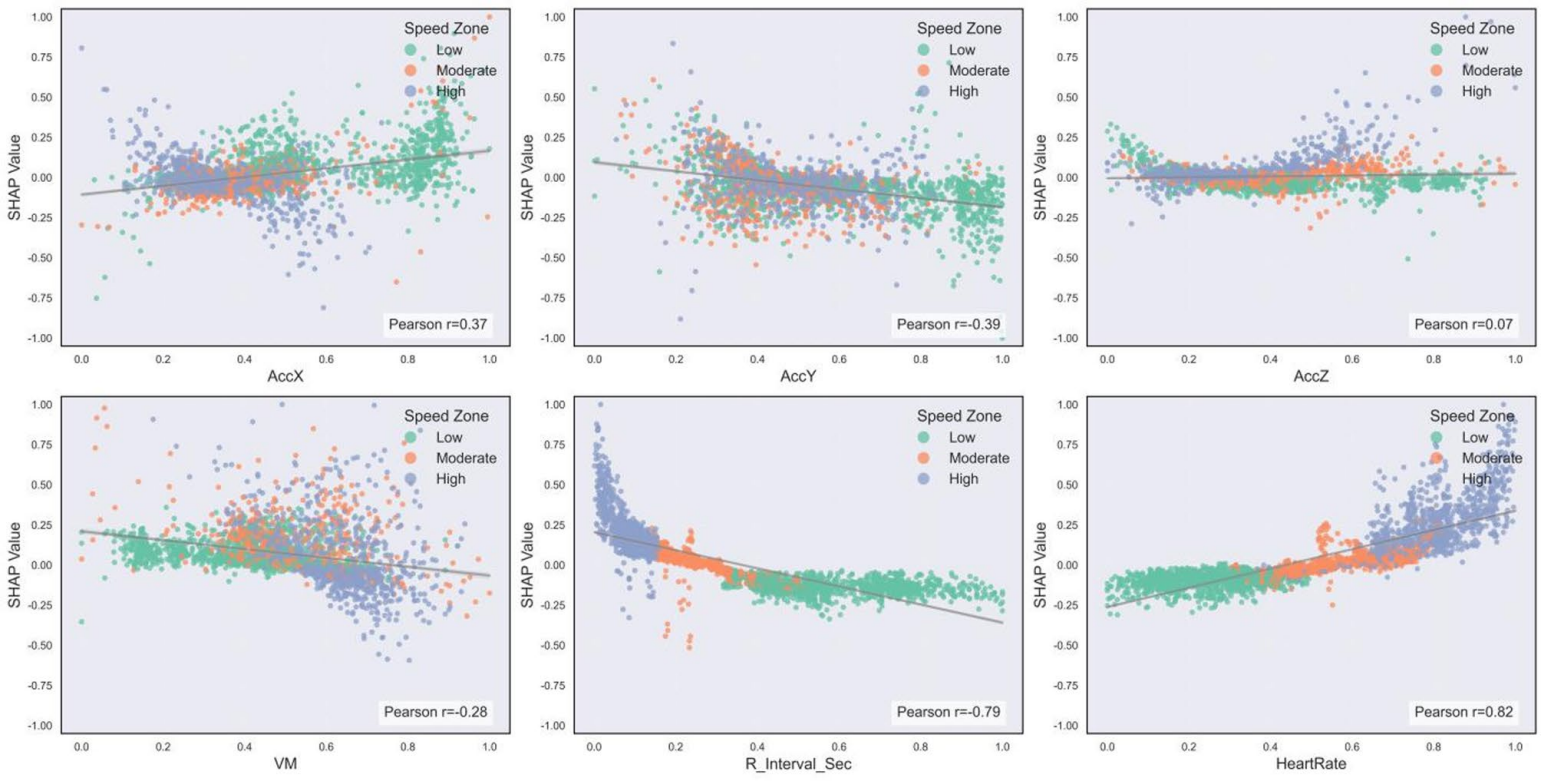
SHAP scatter plot analysis of key base features.

Based on the overall model’s SHAP value analysis, this study selected the triaxial acceleration raw features (AccX, AccY, AccZ) and the acceleration magnitude (VM), representing external load, and fundamental ECG features (R_Interval_Sec, HeartRate), representing internal physiological load, for in-depth analysis. Scatter plots were employed to visualize the association between feature values and their corresponding SHAP values. Point density is indicated by the color gradient, and the fitted trend of feature values and SHAP values is represented by the red line. The Pearson correlation coefficient, as illustrated in Fig. 4, quantifies the direction and magnitude of the relationship between feature values and model contribution. To analyze feature importance across exercise intensities, exercise speed was divided into three intervals: low (3.0–7.5 km/h), medium (7.5–11.0 km/h), and high (11.0–15.0 km/h), based on ACSM criteria and the incremental load settings in the experiment. In the SHAP scatter plot, samples from each interval are color-coded to visualize the trend of feature contributions under varying intensities.

The results showed that AccX was positively correlated with SHAP values (Pearson *r* = 0.37). As the eigenvalues increased, the SHAP values increased, but as the running speed increased, the SHAP values decreased.

In contrast, AccY and VM were negatively correlated with SHAP values (*r* = -0.39 and -0.28). As the eigenvalues increased, the SHAP values decreased. Unusually, AccY and VM showed opposite trends in SHAP values as running speed increased.AccZ, although weakly correlated (*r* = 0.07), still showed a somewhat positive trend in the medium and high intensity intervals. R_Interval_Sec showed a negative correlation with the SHAP values, implying that an increase in the R interval time reduces the contribution to the EE prediction. Heart rate showed the opposite trend. And running speed has the same trend with eigenvalue and SHAP value.

Furthermore, the essence of SHAP values lies in quantifying the marginal contribution of each feature to the prediction model, the extent to which the feature causes a deviation from the baseline prediction. The regions of high point density in the figure, where SHAP values are close to zero, indicate that the model considers these feature value ranges as baseline states. In these ranges, the model relies more on other features, and the fluctuations of these specific feature values have a limited marginal impact on EE prediction. This suggests that the model integrates multiple features to make predictions and is less sensitive to noise in high-density feature value regions.

**Fig. 5:**
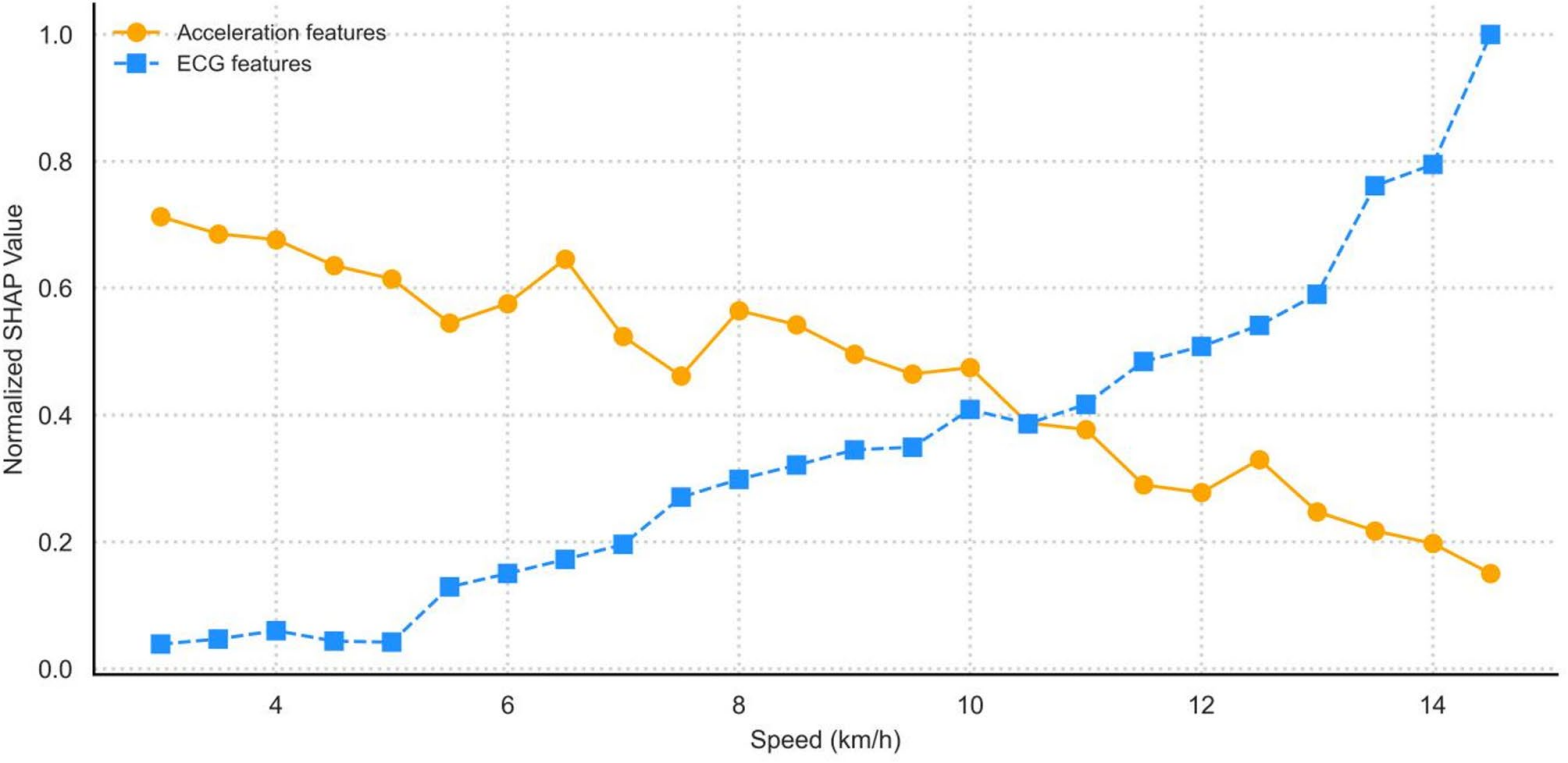
SHAP value analysis of feature contributions across different running speeds.

The patterns and relationships between SHAP values and feature values also reflect that different features contribute differently to the EE model’s predictions at varying exercise speeds. Building upon the above, this study standardized and aggregated the SHAP values of acceleration and ECG features separately and sorted them according to the increasing running speed of the original exercise process. The contribution changes of features to EE prediction across different speed intervals were observed based on SHAP values, as shown in Fig. 5.

In the low-speed phase (3–5 km/h), the contribution of Acc features was maintained at a high and relatively smooth level with a slight decrease, and the contribution of ECG features was at a low level. It indicates that physiological load did not become a dominant factor in the prediction task. As the speed increases (5–10 km/h), the SHAP value of the Acc feature gradually decreases and plateaus, and the contribution of the ECG feature continues to increase. At 10.5 km/h, the intervals of SHAP values for the two types of features intersect, indicating that internal and external loads contribute equally in the prediction task. After 10.5 km/h, the ECG feature contribution increases rapidly and the Acc feature contribution continues to decrease. This During the high-intensity exercise phase, the ECG features became the key drivers of energy expenditure prediction, and the marginal contribution of the Acc features weakened. Using different feature combinations that emphasize various aspects across different speed ranges can lead to more accurate EE estimations. This has important implications for improving exercise monitoring devices and developing personalized exercise prescriptions.

## Discussion

In this study, we proposed a personalized dynamic-static feature fusion framework and analyzed the performance of models constructed with different networks and feature combinations for EE prediction. Firstly, the comparison of R^2^, RMSE, MAE and Bland-Altman plots of LOSO-CV and the test set shows that fusion with features extracted from Acc and ECG signals and choosing CNN + LSTM as the network for processing time series data can effectively improve the model’s prediction performance in terms of EE. This indicates the significance of multi-modal feature fusion and model complexity in addressing time-series problems related to EE. Subsequently, the model with the best predictive performance was selected for global and local SHAP value calculations. Based on these values, we analyzed the overall importance ranking of different features in the EE prediction model, as well as the dynamic relationship between feature contribution, feature values, and running speed.

The proposed EE model integrates time-series networks, multimodal feature fusion (ACC + ECG), and static physiological attributes, and it markedly outperforms conventional approaches. The best configuration, CNN + LSTM-Acc + ECG, achieved R^2^ = 0.886 ± 0.136, RMSE = 0.080 ± 0.056, MAE = 0.062 ± 0.046 in 15-fold LOSO-CV and maintained strong generalisation on an independent test set (R^2^ = 0.906, RMSE = 0.080, MAE = 0.048). Compared with CNN + LSTM-Acc, which only uses acceleration features, RMSE decreases from 0.139 to 0.080, a decrease of 42.4%; MAE decreases from 0.105 to 0.062; and R^2^ improves from 0.617 to 0.886, an increase of 43.6% after fusing ECG. With the same ECG + Acc input, CNN + LSTM reduces the RMSE and MAE by 17.5% and 18.4%, respectively, and improves R^2^ by 3.1% compared to the LSTM model. The results show that fusing the multimodal inputs of external and internal loads helps to capture both exercise intensity and autonomic modulation, which significantly improves the EE prediction accuracy. And the convolutional layer extracts local temporal features before LSTM, which can further enhance the model’s ability to characterise complex nonlinear dependencies.

Traditional methods of estimating energy consumption use non-linear regression^24,25^. But they cannot identify the complex coupling relationship between features and EE. Furthermore, their predictive accuracy is lower in complex or high-intensity exercise scenarios^26^. Machine learning and deep learning models are widely used to improve EE prediction performance. O’Driscoll et al. used acceleration and physiological signals, along with participant characteristics (age, height, weight, and body composition), as input features, combined with machine learning algorithms to build an EE prediction model. They used the relative value of EE, METs, as the output feature, with an RMSE range of 1−1.37 MET, a reduction of approximately 27%^13^. Similarly, Xu et al. combined the acceleration scalar values collected from the wrist with an ANN to construct a prediction model and compared it with linear, logarithmic, and cubic equations. The overall EE prediction error RMSE was between 0.66 and 0.90, a reduction of about 26%^23^. Vibæk et al. used acceleration data from different locations combined with LSTM to build EE prediction models for activities of varying intensities. The optimal model’s correlation and mean absolute percentage error (MAPE) reached 0.883 and 13.9%, respectively, with an R2 value between 0.922 and 0.970^27^. Cortes et al. also built an EE model based on time series. In addition to wearing multiple sensors, they also input personalized features into the model, and the final model’s MAPE was in the range of 5–15%. The above studies used different metrics to evaluate model performance^17^. Although the evaluation metrics vary, they all indicate the effectiveness of multimodal inputs with deep nonlinear modelling in EE prediction. The CNN + LSTM-Acc + ECG model in this study outperforms the above studies in terms of both accuracy and generalisation ability, which further demonstrates that our proposed Dynamic-Static feature fusion framework is able to more accurately capture the complex coupling relationship between different features and EE.

Furthermore, from both the feature and algorithm perspectives, the optimal model constructed in this study was analyzed. Considering that the LSTM network inherently has time-series modeling capabilities, it can capture the temporal information of features. Moreover, different sensor signals exhibit periodic and frequency characteristics. Therefore, the frequency-domain features of acceleration and ECG signals, extracted using FFT and PSD, were combined with the original time-domain features as inputs for the model. Previous studies have shown that both time-domain and frequency-domain features extracted from ECG signals can identify exercise type and intensity. The heart rate variability (HRV) indices, based on the continuous RR intervals, reflect the autonomic nervous system’s regulatory ability, as well as the balance between the sympathetic and parasympathetic systems, with features such as LF and HF being key indicators^28–30^. These features capture physiological signals that reflect the body’s response to both external and internal loads, indirectly representing energy expenditure during exercise at different intensities.

The acceleration signal, recorded through wrist-worn IMUs, directly reflects external mechanical movement and is currently one of the most widely used features for EE prediction. Studies have demonstrated that both time-domain and frequency-domain features extracted from acceleration signals can classify the intensity of different physical activities^31–33^. While the wrist-worn accelerometer in this study is located at the distal end of the body, it is still highly sensitive in capturing movement rhythm and dynamic changes. The frequency-domain features extracted through FFT further uncovered the exercise process information and reflected the overall movement pattern and metabolic level of the body.

In addition, inter-individual physiological variability captured through static attributes such as BMI, percentage body fat and resting heart rate are also important contributors to EE differences. Incorporation of these static attributes could further enhance the personalised predictive power and generalisability of the model across different populations.

From an algorithmic perspective, the LSTM network, with its input, forget, and output gates, is capable of modeling long and short term dependencies in sequences based on both raw and frequency-domain features^34^. The incremental load exercise process in this study has the characteristics of load variation and temporal dependency. The LSTM component enables the model to infer current EE by integrating preceding trends through its gating mechanism. CNN layers extract local feature information within the time steps, identifying high-intensity actions or periodic changes during running. Therefore, the CNN + LSTM network essentially records the dynamic response process of running and EE based on temporal variations.

To further analyze and interpret the contribution of features within the EE model, we used SHAP values to analyze the optimal model. SHAP, which is based on the Shapley value from game theory, calculates the marginal contribution of each feature, providing a clear measure of its importance to the model’s predictions^35^. In the global feature ranking, ECG features such as R_Interval_Sec, HR, and R_Interval_TotalPower, along with acceleration features like AccX_TotalEnergy, AccY_TotalEnergy and VM showed high SHAP values — indicating a strong overall influence on EE prediction. It has been found that R-wave plays an important role in EE prediction task, and this study also extracts the frequency domain features on the R-wave of ECG signals, which also proves the importance of R-wave for EE prediction^36^. R_Interval_Sec and HR directly reflect the body’s metabolic response during physical activity, while frequency-domain features like LF, HF, and R_Interval_TotalPower capture the balance between sympathetic and parasympathetic nervous system activity. Research has shown a strong correlation between physical activity EE and vagal tone, which is supported by the importance of R_Interval_Sec, HF, and LF features in our SHAP analysis^37^. Physiologically, energy expenditure (EE) is derived from oxygen uptake (VO_2_) and carbon-dioxide production (VCO_2_). During incremental exercise, the oxygen demand of working muscles rises sharply. Sympathetic activation releases noradrenaline, which increases sinoatrial pacing and myocardial contractility, elevating heart rate (HR) and stroke volume (SV) simultaneously. The resulting rise in cardiac output (CO = HR × SV) accelerates systemic circulation and augments oxygen delivery. Thus the frequency domain indicators of the R-wave and ECG are a direct reflection of the EE of the body’s motor processes^38–40^.

The frequency-domain features of acceleration signals can reflect arm swing amplitude and stride frequency during running, enabling accurate estimation of instantaneous running speed. The SHAP value ranking of features extracted via FFT, such as AccX_TotalEnergy and AccY_TotalEnergy, demonstrates that the temporal dynamics of acceleration and the amplitude of high order signal fluctuations during running also play a crucial role in EE prediction. This finding aligns with the work of Xiao et al., who similarly highlighted the importance of multi-scale motion process frequency-domain information, which provides a supplement to motion rhythmicity based on dynamic time modeling^41,42^. Overall, the SHAP-based importance ranking supports the effectiveness of combining ECG and acceleration frequency features to jointly capture internal physiological load and external mechanical demand during dynamic exercise.

Furthermore, the relationship between feature value changes and model predictions was further elucidated through scatter plots of local SHAP values, and the changes in feature contributions were analyzed based on SHAP values at different running speeds. During low-intensity exercise, there were issues of overestimation or underestimation in EE estimation using acceleration or heart rate^43^. In moderate-intensity exercises such as walking and jogging, the accuracy of EE calculation using acceleration features or heart rate features was high^44^. In this study, incremental load exercise started from 3 km/h, where acceleration features contributed more to EE prediction than ECG features. The model relied more on acceleration features for prediction, with ECG features serving a supplementary role; during this phase, cadence, arm swing amplitude, and ECG changes were relatively stable. As running speed increased, the model’s reliance on ECG features for prediction increased; at 10.5 km/h, the contribution of ECG features surpassed that of acceleration features, and the model relied more on ECG features for prediction. This reflects the fact that during the medium to high intensity exercise phase, after the exercise intensity reaches a certain threshold, the body’s EE is mainly limited by cardiorespiratory function and physiological load, and the ECG becomes the main feature predicted by the model. During incremental loading exercise, as the exercise intensity increases, skeletal muscle demand for oxygen and substrates increases, sympathetic nerves release norepinephrine, and heart rate and myocardial contractility increase^39,45^. Analysis of the above SHAP values and feature values revealed that the fusion of multi-modal features of acceleration and ECG at different exercise intensity phases could improve the accuracy of EE prediction, with different features playing a complementary role in model prediction.

## Conclusion

This study proposed a personalized dynamic-static feature fusion framework for predicting EE during incremental load exercise. The CNN + LSTM model integrating tri-axial acceleration and ECG features demonstrated superior predictive accuracy compared to conventional autoregressive (linear) models and single-modal LSTM models. This improvement can be attributed to the CNN layers’ ability to extract local temporal patterns and the LSTM layers’ capability to capture temporal dependencies across longer intervals, effectively modeling complex physiological dynamics. Furthermore, incorporating static individual characteristics such as BMI, body fat percentage, resting heart rate, and resting VO_2_ significantly enhanced model personalization and reduced inter-individual prediction errors. Additionally, SHAP values based on the optimal model were used to further analyze both global and local feature importance. During moderate to high intensity exercise, acceleration features play a dominant role in EE prediction, with the contribution of ECG features gradually increasing as exercise intensity increases. In the high intensity exercise phase, ECG features become the dominant predictor, demonstrating the complementary and dual contributions of the two feature types in EE prediction at different exercise intensities. In conclusion, this study provides a feasible framework for EE prediction, which provides a theoretical foundation and practical reference for related studies such as exercise load monitoring, personalised exercise plan development and exercise performance analysis. In the future, the model will be tested in a wider range of populations to further improve the feasibility of applying the model in different populations and integrating it into real-time wearable devices.

## Research limitations

Despite the favourable predictive results achieved in this study, several limitations remain. First, the small sample size (*n* = 19) and relative lack of diversity in terms of participants’ age, gender, and health status in this study may limit the model’s ability to generalise to a wider population. Therefore, future studies need to include a more diverse and large sample population to improve the external validity and generalisation performance of the model.

Second, the Weir equation (based on VO_2_ and VCO_2_ measurements) was used as the reference standard for energy expenditure in this study. Although this method is widely accepted under steady-state exercise conditions, it relies on a fixed protein oxidation rate as well as steady-state metabolic assumptions, which may not be fully applicable during the dynamic incremental loading exercise or the short 10-second window used in this study. As a result, a degree of error or fluctuation may be introduced during the high intensity exercise phase. Follow-up studies may consider exploring metabolic models or modified formulas that are more suitable for dynamic exercise processes to further improve the accuracy of energy expenditure estimation.

## Data Availability

The dataset generated during this study is not publicly available; however, it can be obtained from the first author upon reasonable request.

## Code availability

The code used in this study is available from the first author upon reasonable request.
